# Optimization of ^99m^Tc whole‐body SPECT/CT image quality: A phantom study

**DOI:** 10.1002/acm2.13528

**Published:** 2022-01-20

**Authors:** Mansour M. Alqahtani, Kathy P. Willowson, Chris Constable, Roger Fulton, Peter L. Kench

**Affiliations:** ^1^ Faculty of Medicine and Health University of Sydney Sydney Australia; ^2^ Department of Radiological sciences College of Applied Medical Science Najran University Najran Saudi Arabia; ^3^ Department of Nuclear Medicine Royal North Shore Hospital Sydney Australia; ^4^ Institute of Medical Physics Faculty of Science The University of Sydney Sydney Australia; ^5^ HERMES Medical Solutions, Strandbergsgatan 16 Stockholm Sweden; ^6^ Department of Medical Physics Westmead Hospital Sydney Australia

**Keywords:** acquisition time, background variability, CNR, contrast recovery, image reconstruction, SPECT/CT

## Abstract

**Purpose:**

Investigate the impact of acquisition time and reconstruction parameters on single‐photon emission computed tomography/computed tomography (SPECT/CT) image quality with the ultimate aim of finding the shortest possible acquisition time for clinical whole‐body SPECT/CT (WB‐SPECT/CT) while maintaining image quality

**Methods:**

The National Electrical Manufacturers Association (NEMA) image quality measurements were performed on a SPECT/CT imaging system using a NEMA International Electrotechnical Commission (IEC) phantom with spherical inserts of varying diameter (10–37 mm), filled with ^99m^Tc in activity sphere‐to‐background concentration ratio of 8.5:1. A gated acquisition was acquired and binned data were summed to simulate acquisitions of 15, 8, and 3 s per projection angle. Images were reconstructed on a Hermes (HERMES Medical Solutions AB, Stockholm, Sweden) workstation using eight subsets and between 4 and 24 iterations of the three‐dimensional (3D) ordered subset expectation maximization (OSEM) algorithm. Reconstructed images were post‐smoothed with 3D Gaussian filter ranging from 0 to 12 mm full‐width at half maximum (FWHM). Contrast recovery, background variability, and contrast‐to‐noise ratio were evaluated

**Results:**

As expected, the spheres were more clearly defined as acquisition time and count statistics improved. The optimal iteration number and Gaussian filter were determined from the contrast recovery convergence and level of noise. Convergence of contrast recovery was observed at eight iterations while 12 iterations yielded stabilized values at all acquisition times. In addition, it was observed that applying 3D Gaussian filter of 8–12 mm FWHM suppressed the noise and mitigated Gibbs artifacts. Background variability was larger for small spheres than larger spheres and the noise decreased when acquisition time became longer. A contrast‐to‐noise ratio >5 was reached for the two smallest spheres of 10 and 13 mm at acquisition times of 8 s

**Conclusion:**

Optimized reconstruction parameters preserved image quality with reduce acquisition time in present study. This study suggests an optimal protocol for clinical ^99m^Tc SPECT/CT can be reached at 8 s per projection angle, with data reconstructed using 12 iterations and eight subset of the 3D OSEM algorithm and 8 mm Gaussian post‐filter.

## 1 INTRODUCTION

Single‐photon emission computed tomography (SPECT) combined with X‐ray computed tomography (SPECT/CT) yields information on both physiology (SPECT) and anatomy (CT).^1^ Recently, advances in SPECT/CT hardware and software have fueled SPECT/CT's ongoing clinical development. This includes the application of whole‐body (WB) SPECT/CT whereby multiple consecutive axial fields‐of‐view (AFOV) are stitched together, yielding three‐dimensional (3D) WB images analogous to those obtained from positron emission tomography–computed tomography (PET/CT). This technique has the potential to replace WB planar scintigraphy in, for example, WB bone scanning.^2^, ^3^ WB‐SPECT/CT can be clinically useful in increasing diagnostic confidence when evaluating bone metastases (BM) and when assessing suspicious or equivocal lesions via WB planar scintigraphy in cancer cases, as attested by a number of studies.^4^, ^5^, ^6^

Considerable progress has been made in SPECT image reconstruction with the advancement of a variety of iterative reconstruction schemes that integrate correctional techniques to model system response, photon scattering, and attenuation.^7^, ^8^ Due to these developments, SPECT image quality has improved with regard to contrast, noise minimization, resolution, and image quantification,^9^ aiding patient follow up and interpatient comparisons. Spatial resolution of PET is typically twice as good as SPECT and count sensitivity is 20–30 times higher.^10^, ^11^, ^12^ Although the spatial resolution and sensitivity of SPECT are poorer than PET, SPECT does have some advantages. SPECT radionuclides typically have a longer physical half‐life that is better matched to the biologic half‐lives of physiological processes being imaged. SPECT imaging also enjoys greater availability of radiotracers without the necessity of a fast distribution network or a cyclotron as required for PET, the ability to undertake simultaneous multi‐tracer studies in the same imaging session, as well as being generally less costly and having a more widely installed base of SPECT/CT systems.^11^, ^13^

Related to the lower sensitivity of SPECT compared to PET is the issue of generally longer scan acquisition times for SPECT. Longer scan time negatively affects patient throughput and reduces patient comfort and compliance, resulting in greater motion during the study. For this reason, clinical SPECT examinations with dual head cameras are often limited to a single AFOV of approximately 50 cm over targeted organs such as the heart or brain, with typical SPECT acquisition times ranging from 20 to 30 min.^14^ In the case of WB bone scanning, single AFOV SPECT is often used to complement planar WB images over a specific axial area of interest rather than applied to the entire body as multiple stitched AFOVs. Reducing the acquisition time per AFOV is therefore likely to reduce motion artifacts,^15^ improve patient's comfort and throughput,^1^, ^2^ and it is also a prerequisite for the wider adoption of WB‐SPECT imaging.

The possibility of performing SPECT over a shorter period is technically attainable but should not reduce image quality. Reduce acquisition time has been investigated in the specific application of myocardial perfusion imaging using SPECT in various studies.^16^, ^17^, ^18^ Ali et al.^19^ investigated the used of half‐time and full‐time myocardial perfusion images reconstructed with resolution recovery (RR) on 112 patients. They reported that there was no difference in terms of image quality between full‐time and half‐time gated images as well as no significant differences in quantitative analysis of left ventricle volume and function.

A limited studies have assessed a range of compensation approaches to reduce acquisition time and improve image quality in bone SPECT/CT imaging. RR applied to SPECT image reconstruction, based on a 3D model of collimator and system resolution, can increase not just spatial resolution but also SPECT image quality even when acquisition time was halved.^20^, ^21^ However, these studies have mainly focused on novel methods for image reconstruction. Another prospective study reported the possibility to perform 3‐min “ultra‐fast” (UF)‐SPECT/CT for BM assessment without compromising diagnostic confidence. However, the reconstruction algorithm parameters were not optimized. Moreover, their UF‐SPECT/CT protocol was employed as a single AFOV SPECT/CT complementary to the WB planar scan, whereas our ultimate aim is to replace the planar scan altogether with WB‐SPECT/CT alone.

Investigation of the reduction of image acquisition times cannot be done using repeat measurements on the same human subject because of the additional imaging time burden. Radioactive and biological decay of the radiotracer can also confound the results of such repeated measures. Radioactive decay and camera time limitations are also applicable to phantom studies. Therefore, techniques to re‐sample the acquisition data into variable time ranges is needed. Image gating, which most modern SPECT cameras support, can be exploited to study the effects of reduced acquisition times in a variety of imaging scenarios. This has been demonstrated by Bailey and Kalemis,^22^ using an electrocardiography (ECG) simulator as an external trigger to perform non‐physiological gating to yield almost identical yet statistically distinct partitioned datasets to investigate acquisition time reduction. Data partitioning helps to establish the ideal or appropriate length of time for a scan, the count rate required, and activity administered to attain suitable quality of images. Such an approach can be implemented on most imaging systems without alterations, since the majority of installed gamma cameras do not have a list mode acquisition capability.

In the present study, the above described gated methodology was used to generate SPECT scans of differing shorter acquisition durations based on a single standard duration acquisition. We therefore investigate the impact of acquisition time and reconstruction parameters on image quality of ^99m^Tc SPECT/CT. Using a National Electrical Manufacturers Association and the International Electrotechnical Commission (NEMA IEC) body phantom and clinical examples, we investigated the optimal parameters for both acquisition and reconstruction, with the goal of reducing acquisition time for WB‐SPECT/CT without detrimental effects on lesion detection or quantification.

## 2 MATERIALS AND METHODS

### 2.1 Phantom experiment design

Performance evaluation for image quality was carried out with the NEMA IEC body phantom. This phantom comprises a fillable torso cavity that can encompass up to six fillable spheres of various internal diameters (37, 28, 22, 17, 13, and 10 mm).

A solution of ^99m^Tc in a water volume of 1200 ml was prepared with ∼ 214.2 MBq. From this solution ∼48 ml volume was withdrawn for the six spheres. Injection of activity in the spheres (37, 28, 22, 17, 13, and 10 mm, respectively) involved placing the syringe catheter tip through the removed filler cap opening with a narrow tube from the external lid side. The remaining ∼1152 ml of solution was diluted in the 9787 ml background compartment volume so that the sphere‐to‐background concentration ratio was 8.5:1 achieving a concentration in spheres and background of 178.5 and 21 kBq/ml, respectively. The activity concentration level in the phantom background was chosen to be similar to that obtained in a clinical study of patients undergoing ^99m^Tc‐methylene diphosphonate (MDP) bone SPECT/CT imaging.^11^, ^23^ To accurately determine the measurement for both the background and spheres compartments of the phantom at the scan time, the aliquots from both compartments were measured and compared with standard in an auto‐gamma counter (2480 Wizard2, PerkinElmer, Waltham, MA, USA).

### 2.2 Data acquisition and image gating

A dual head SPECT/CT system (Symbia Intevo.6, Siemens Healthineers, USA) equipped with a low‐energy high‐resolution collimator was employed for data collection. The local clinical bone SPECT/CT acquisition protocol was used, specifically: a 128 × 128 matrix size, with 60 views acquired per detector (120 projections in total), of 15 s/view, over 360° rotation within a non‐circular patient contoured orbit in step‐and‐shoot mode. The acquisition pixel size was 4.8 mm in both *x* and *y* directions.

The image data were acquired using simulated gating into 15 time bins. An electronic ECG simulator was used to generate a regular R‐wave ECG trigger signal, which was then connected to the ECG input on the SPECT camera. The ECG was used purely to provide a simulated signal to the scanner to allow data to be acquired in a gated mode such that bins could later be summed to various degrees to provide multiple different “acquisition durations” from a single scan. This method has been presented in the literature by Bailey and Kalemis^22^ and is a very useful way of acquiring data to test different count statistics without the need for multiple acquisitions and so lengthy scanner times and avoiding radionuclide decay. The data from each projection were summed to simulated various acquisition times (Table 1). The gated acquisition data were re‐sampling into different acquisition durations with in‐house software using Interactive Data Language Program (Research Systems International, Boulder, CO, USA). From this, data corresponding to projection times of 15, 8, and 3 s were generated for reconstruction and analysis.

**TABLE 1:** Summary of the simulating shorter acquisition time using gated data bins

Gated bin data	1–3	1−8	1−15
Acquisition time simulation (s)	3	8	15^a^

^a^ Identical to the local non‐gated acquisition protocol.

### 2.3 Image reconstruction

SPECT data were reconstructed using a 3D ordered subset expectation maximization (OSEM) algorithm (Hybrid Reconstruction version 3.2, HERMES Medical Solutions AB, Stockholm, Sweden). This program applies RR using a distance‐dependent 2D Gaussian collimator‐detector model, attenuation correction (AC) based on a CT‐derived linear attenuation map, and scatter correction using the attenuation map and a Monte Carlo simulation algorithm.^24^ Reconstructions were performed using iteration numbers ranging from 4 to 24, with a constant subset number of eight and post‐reconstruction 3D Gaussian filters ranging from 0 to 12 mm full‐width at half maximum (FWHM). The effect of these parameters on the contrast recovery (RC) and background variability (BV) was examined, with the ultimate aim of finding the shortest possible acquisition time for WB‐SPECT/CT while maintaining acceptable image quality.

The expected final spatial resolution of the SPECT images was approximately 10–12 mm FWHM. Although it would be possible to reduce the image noise by increasing image slice thickness (or in‐plane pixel size), the images were reconstructed in the original pixel dimensions of 4.8 mm x 4.8 mm x 4.8 mm to preserve the spatial resolution of the original data.

### 2.4 Image analysis

**FIGURE 1:**
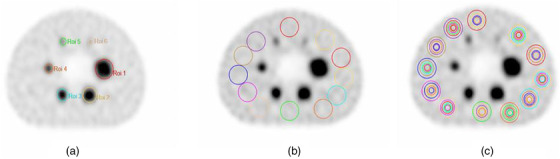
Image quality analysis: (a) regions of interest (ROIs) on hot spheres; (b) background ROIs; (c) background ROIs for all diameters (37, 28, 22, 17, 13, and 10 mm)

The process of image analysis began with the selection of a central slice through the middle of the hot spheres, as well as the selection of four axial slices, two in each direction relative to the middle slice. As shown in Figure 1a, circular regions of interest (ROIs) were delineated on each of the six hot spheres. Subsequently 12 ROIs of identical size were delineated in the background of the phantom on the center slice, according to NEMA specifications.^25^ Figure 1b illustrates the 12 ROIs with a diameter of 37 mm that were delineated in the phantom background so that the distance between the ROIs and the phantom margin was 15 mm and the distance to the other hot spheres was no less than 15 mm. Furthermore, smaller sphere ROIs (28, 22, 17, 13, and 10 mm) were delineated concentrically within the 37 mm diameter ROIs. Sphere delineation on the other four chosen slices was performed identically (Figure 1c). Hence, a total of 60 ROIs of each size were delineated on the background in line with the NEMA NU 2‐2018 standard.

The RC for each hot sphere, s, was determined using Equation (1), where *C*_H, s_ is the mean pixel value in the ROI for sphere s, *C*_B, s_ is the mean value of the background ROI counts for sphere s, and *R* is the ratio of the true activity concentration in the hot spheres to the true activity concentration in the background. The percent BV for each sphere was calculated according to Equation (2), where SD_s_ is the standard deviation of the background ROIs counts for sphere s, calculated using Equation (3). Contrast‐to‐noise ratio (CNR), a measure of image quality, which is not a standard NEMA parameters, was calculated for each sphere from Equation (4) according to Refs.,^26^, ^27^ where SD_ROI_ is standard deviation of the value in the background ROI. The CNR was intended to demonstrate object detectability based on the Rose criterion (CNR > 5).^27^, ^28^ GraphPad Prism 9.0 (GraphPad Software, Inc., CA, USA) was used for graphical analysis.

(1) $$RC_{s}=\frac{\left(C_{H,s}/C_{B,s}\right)-1\;}{R-1}\times 100$$

(2) $$BV_{s}=\frac{SD_{s}}{C_{B,s}}\times 100$$

(3) $$SD_{s}=\sqrt{\sum_{k=1}^{K}\frac{{\left(C_{B,s,k\;}-\;C_{B,s}\right)}^{2}}{K-1}{\;}_{\;}}\;$$

where the sum is taken over the *K* = 60 background ROIs.

(4) $$\mathrm{CN}R_{s}=\frac{C_{H,s}-C_{B,s}\;}{SD_{\mathrm{ROI}}}$$

### 2.5 Clinical study example

This study was approved by the Northern Sydney Local Health District Human Research Ethics Committee (approval no. 2020‐02150). The SPECT/CT reconstruction approach was applied to two clinical bone studies using a gating technique. The studies were acquired in the Department of Nuclear Medicine, Royal North Shore Hospital. A single FOV bone SPECT/CT was acquired covering the knees in patient 1 and covering the lumbar/thoracic spine in patient 2.

### 2.6 Clinical study analysis

The noise characteristics, contrast, and CNR were evaluated for each acquisition time using statistics obtained from ROIs in both normal and abnormal areas in bone.^26^, ^29^, ^30^ ROIs were drawn on the hot abnormal bone area identified by the reporting physician. Additional ROIs were delineated over areas of adjacent normal bone guided by the CT boundaries of the fused SPECT/CT images.

## 3 RESULTS

### 3.1 Phantom study

#### 3.1.1 Contrast recovery and background variability

**FIGURE 2:**
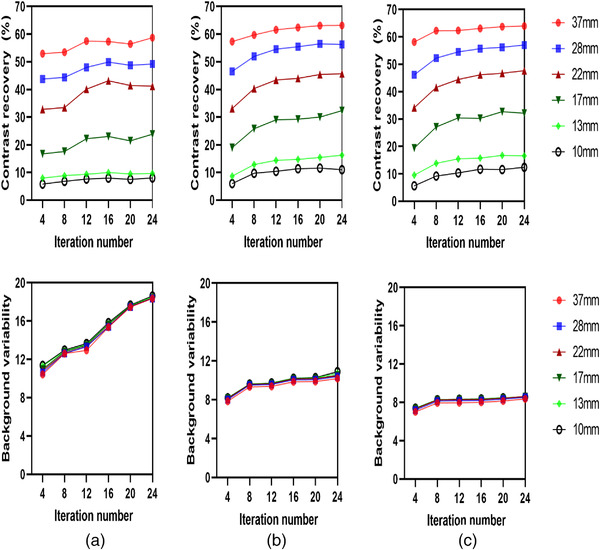
Contrast recovery and background variability as a function of iteration number: (a) 3 s/view, (b) 8 s/view, and (c) 15 s/view. An 8 mm Gaussian filter and eight subsets were used for all reconstructions

The effect of the number of OSEM iterations for various acquisition times is provided in Figure 2. As anticipated, there was an increase in RC with an increasing number of iterations at the expense of high BV. Furthermore, there was an increase in RC proportional to sphere size. RC convergence was achieved at 12 iterations with less than 3.5% of the variation. At 12 iterations the contrast value for the 37, 28, 22, 17, 13, and 10 mm spheres were 62.3%, 54.5%, 44.4%, 30.4%, 15.4%, and 10.3% at 15 s/view; 61.5%, 54.5%, 43.4%, 29%, 14.4%, and 10.3% at 8 s/view; and 57.4%, 47.9%, 40.1%, 22.4%, 9.3%, and 7.6% at 3 s/view, respectively (see Table S1).

BV for small spheres was higher compared to larger spheres, which is essentially noise. As expected, greater acquisition time resulted in a noise reduction. Figure 2 illustrates image noise as a function of iteration number at various acquisition times while other reconstruction parameters remained constant, for example, eight subsets and 8 mm FWHM Gaussian filter. In the 37 mm sphere, image noise rose from 7.84% to 10.17% when iterations were increased from 4 to 24 iterations at 8 s/view and from 7.01% to 8.35% at 15 s/view. Meanwhile, in the 10 mm sphere, image noise rose from 8.19% to 10.89% when iterations were increased from 4 to 24 at 8 s/view and from 7.39% to 8.56% at 15 s/view. Furthermore, image noise at 3 s/view rose from 10.42% to 18.36% and from 11.38% to 18.63% for the 37 and 10 mm spheres, respectively (Table S1).

**FIGURE 3:**
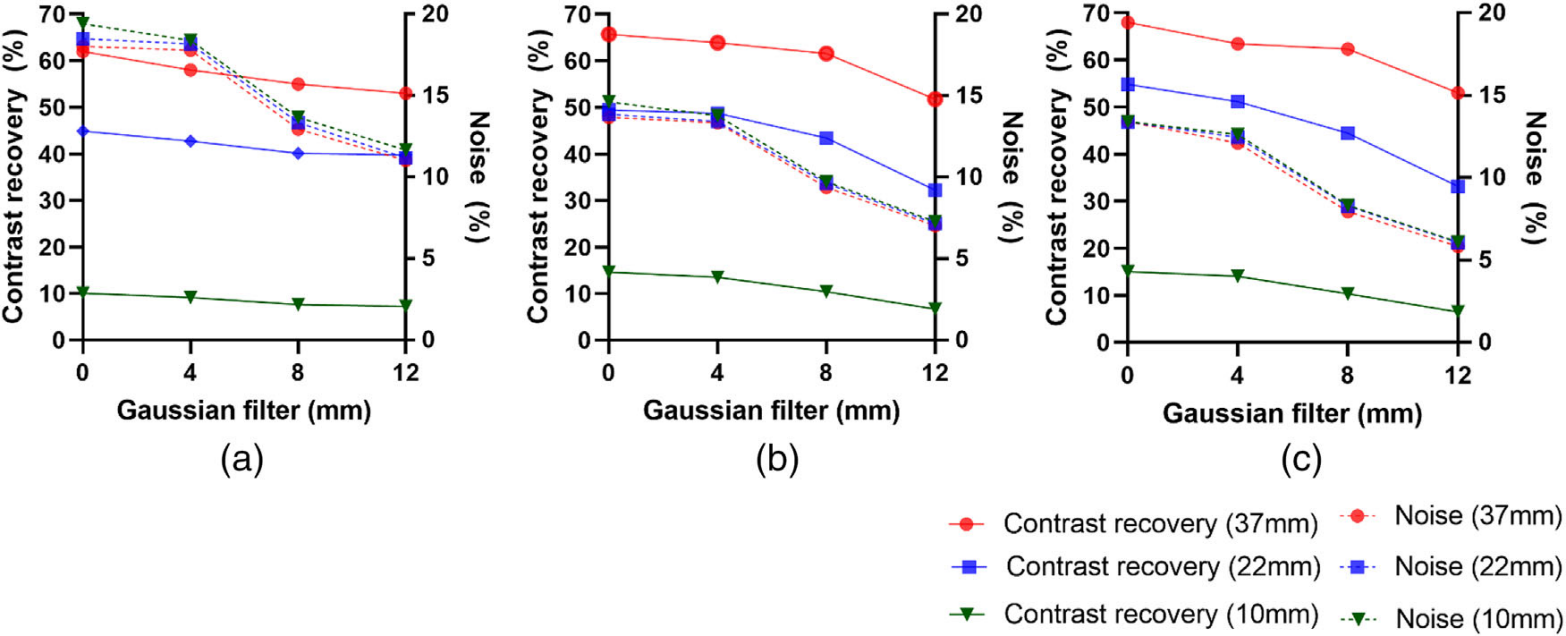
Relation between contrast recovery and the noise as function of Gaussian filter full‐width at half maximums (FWHMs) (0–12 mm) for largest (37 mm), medium (22 mm), and smallest (10 mm) spheres: (a) 3 s/view, (b) 8 s/view, and (c) 15 s/view. Twelve iteration and eight subsets were used for all reconstructions

**FIGURE 4:**
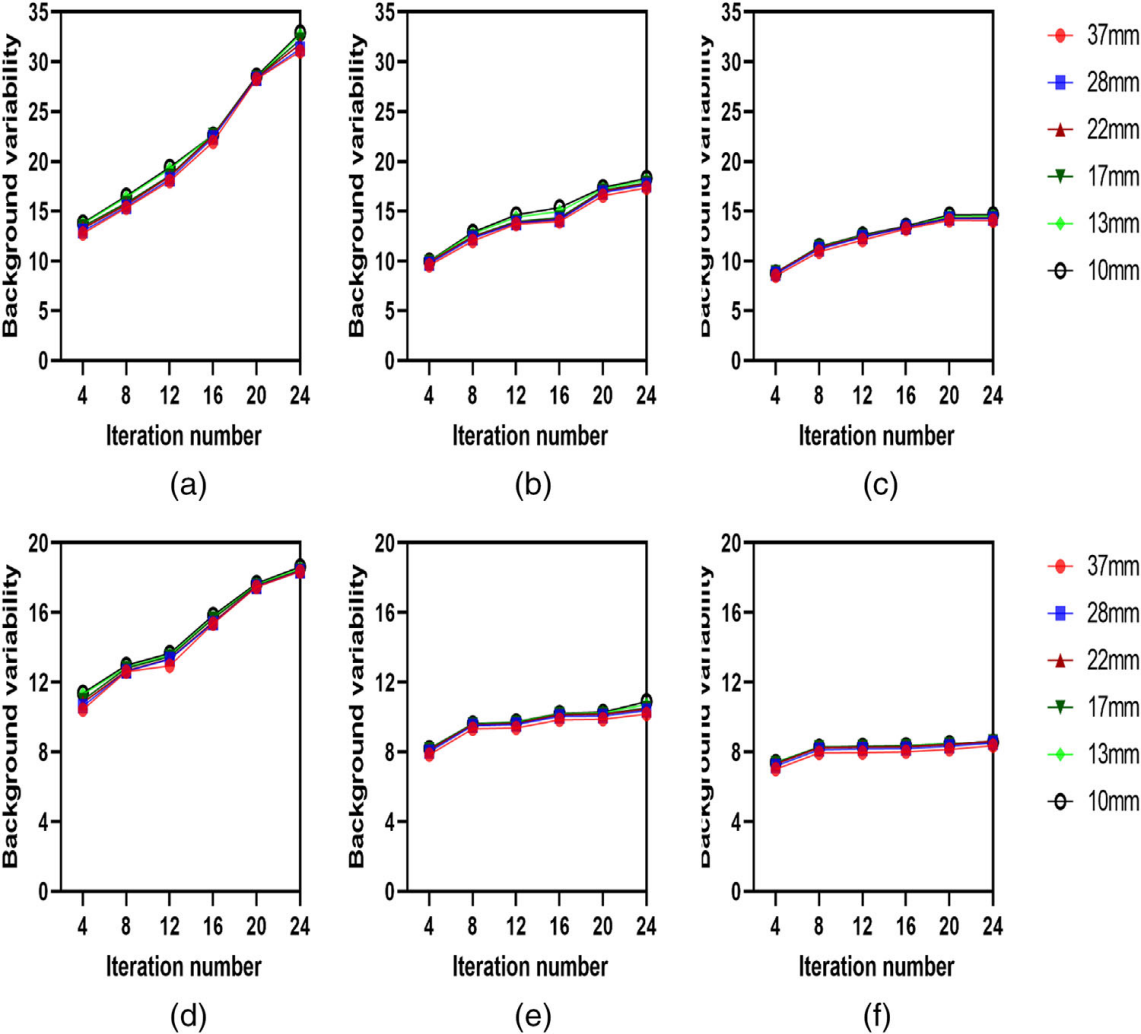
Background variability as a function of the number of iterations in spheres of different size. Comparison of non‐Gaussian filter (a–c) versus a 8 mm full‐width at half maximum (FWHM) Gaussian filter (d–f) for three different acquisition times: 3 s (a and d), 8 s (b and e), and 15 s (c and f)

Figure 3 shows the effect of Gaussian filter width on RC and BV for the 10, 22, and 37 mm diameter spheres. The 10 mm sphere has a higher variability value (higher image noise) than the larger 22 and 37 mm spheres. For all acquisition times, the noise level reduced as the Gaussian filter width was increased, with similar noise levels for filter widths of 0 and 4 mm. RC improves when no Gaussian post‐filter is applied, which is particularly significant for the small spheres that are poorly seen due to limits in spatial resolution. At 15 s/view, the RC percentage increased 45% higher for the 10 mm sphere than the larger spheres, for example, 23% improvement for the 22 mm sphere when Gaussian filter FWHM decreased from 8 to 0 mm. At 8 s/view, the RC increased 40% higher for the 10 mm sphere than the larger spheres, for example, 14% improvement for the 22 mm sphere when Gaussian filter FWHM decreased from 8 to 0 mm. Also, at 3 s/view, the RC increased 32% higher for the 10 mm sphere compared to the larger spheres, for example,12% improvement for the 22 mm sphere when Gaussian filter FWHM decreased from 8 to 0 mm (see Tables S2 and S3). However, the BV appears to be a considerably higher value in images reconstructed without an FWHM Gaussian filter than in images reconstructed using an 8 mm FWHM Gaussian filter. Furthermore, higher noise levels were observed with shorter acquisition scan times at 3 s/view (Figures 4 and S1). Therefore, there is a trade‐off between image spatial resolution and noise.

#### 3.1.2 Optimizing reconstruction of lesion detection CNR at different acquisition time

**FIGURE 5:**
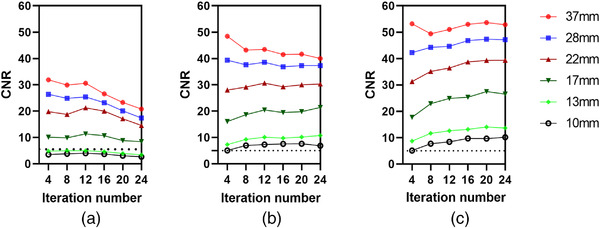
Contrast‐to‐noise ratio (CNR) as a function of iteration number for all sphere sizes and three different acquisition times: (a) 3 s/view, (b) 8 s/view, and (c) 15 s/view. The dotted line in each figure represents CNR = 5 (Rose criteria). An 8 mm Gaussian filter and eight subsets were used for all reconstructions

**FIGURE 6:**
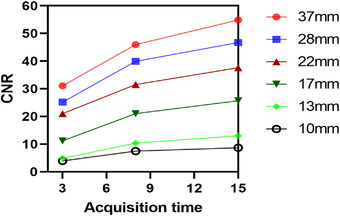
Contrast‐to‐noise ratio (CNR) for increasing acquisition time for all different sphere sizes. An 8 mm Gaussian filter and eight subsets were used for all reconstructions

CNR can serve as the basis for quantitatively measuring the visibility of specific uptake in the image.^31^ Figure 5 provides plots of CNR (calculated from Equation (3)) as a function of iteration number for different acquisition times and sphere diameters. These data are also listed in Table 2. As indicated by these plots, the larger the size of the regions, the better CNR at all acquisition times, as can be seen with 37 mm spheres (red lines) compared with 10 mm spheres (black line). Also, the two smallest spheres of 10 and 13 mm are “visible” in accordance with the Rose criterion (horizontal dotted line) at acquisition times greater than or equal to 8 s/view, with visibility increasing with the iteration number. Additionally, the CNR was overall better with lengthier acquisitions (Figure 6).

**TABLE 2:** Contrast‐to‐noise ratio (CNR) as a function of iteration number at different acquisition times for all spheres

	CNR																	
	3 s/view						8 s/view						15 s/view					
Iteration number	10 mm	13 mm	17 mm	22 mm	28 mm	37 mm	10 mm	13 mm	17 mm	22 mm	28 mm	37 mm	10 mm	13 mm	17 mm	22 mm	28 mm	37 mm
4	3.54	4.82	10.12	19.79	26.40	31.90	5.05	7.30	16.07	28	39.33	48.45	5.14	8.70	17.76	31.28	42.25	53.17
8	3.82	4.95	9.85	18.75	24.83	29.89	6.97	9.25	18.65	29.16	37.60	43.20	7.73	11.67	22.89	35.14	44.27	49.41
12	4.02	4.96	11.39	21.28	25.42	30.61	7.30	10.14	20.45	30.64	38.55	43.46	8.40	12.60	24.89	36.39	44.65	51.04
16	3.71	4.64	10.68	20	23.19	26.56	7.55	9.77	19.45	29.25	36.86	41.47	9.69	13.16	25.34	38.73	46.79	53
20	3.08	3.91	8.82	17.10	20.09	23.31	7.64	10.18	19.80	30	37.31	41.65	9.66	14.04	27.47	39.31	47.36	53.65
24	2.67	3.41	8.41	14.55	17.39	20.81	6.88	10.77	21.43	30.31	37.30	39.98	10.18	13.64	26.53	39.38	47.16	52.84

*Note*: An 8 mm Gaussian filter and eight subsets was used for all reconstructions.

**FIGURE 7:**
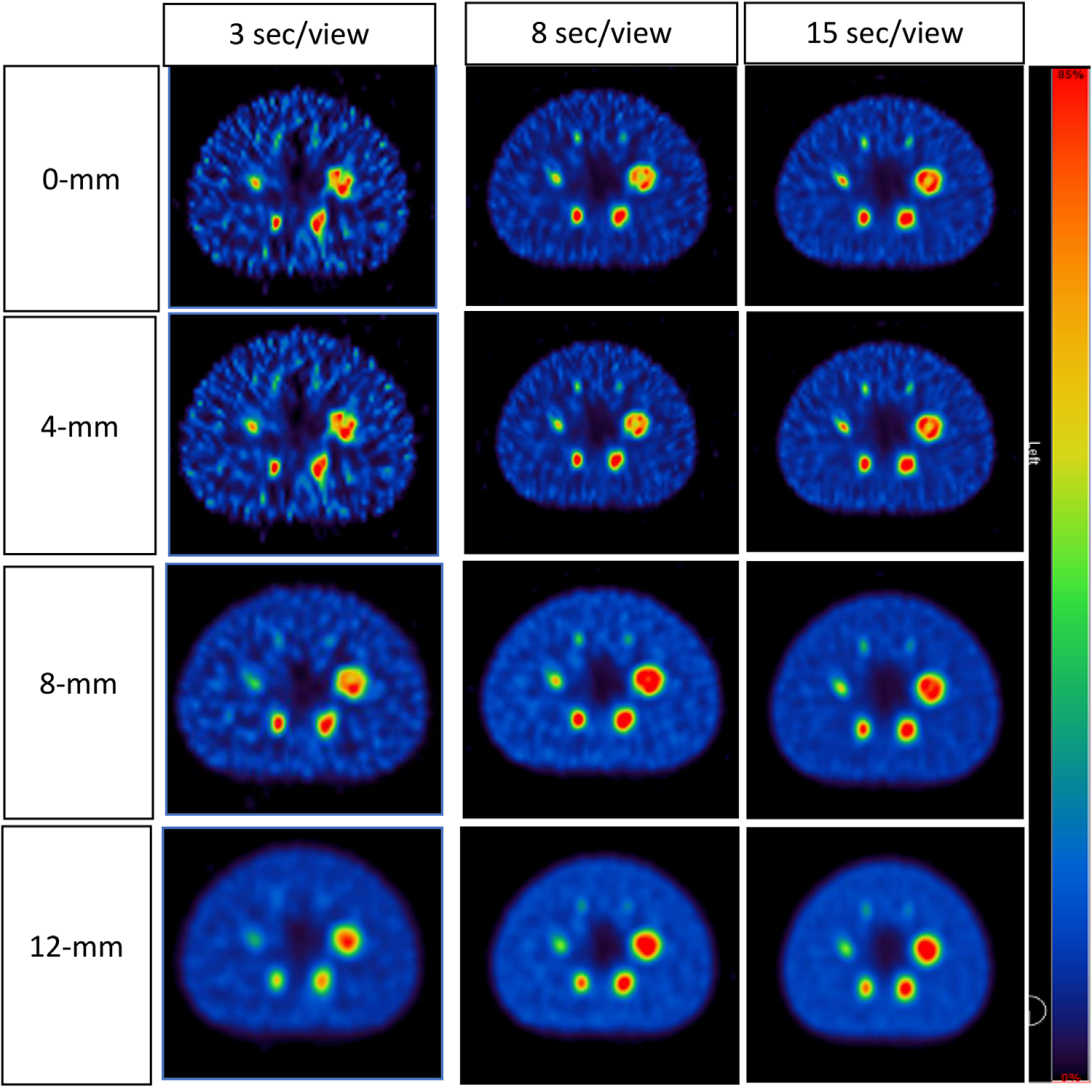
Reconstructed images with varying filter widths and acquisition times. Twelve iterations and eight subsets were used for all reconstructions

Figure 7 demonstrates the impact of the Gaussian filter of 0–12 mm FWHM at different acquisition times. Subjectively the background image noise is reduced with increasing Gaussian filter FWHM. At the same time, the image blurring effect of the Gaussian post‐filter can be appreciated with reference to the smallest sphere. This figure illustrates the inherent trade‐off between spatial resolution and noise that must be made when selecting a Gaussian post‐filter. It can also be observed that the smallest sphere is not visible at the lowest acquisition time at 3 s regardless of the post‐filter applied.

### 3.2 Clinical imaging

**FIGURE 8:**
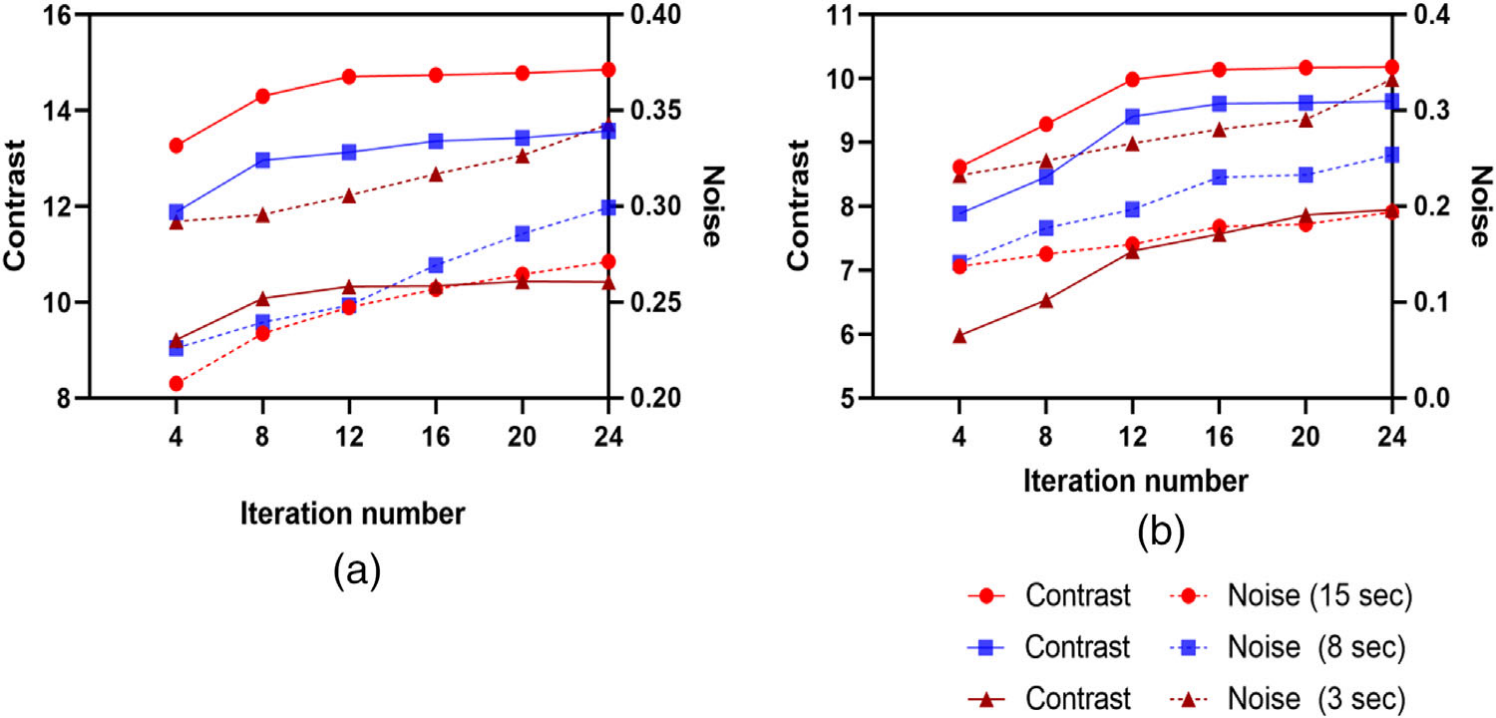
Relation between contrast and the noise as a function of iteration number for the single‐photon emission computed tomography/computed tomography (SPECT/CT) images in patient 1 (a) and patient 2 (b) for three different acquisition times. An 8 mm Gaussian filter and eight subsets were used for all reconstructions

**FIGURE 9:**
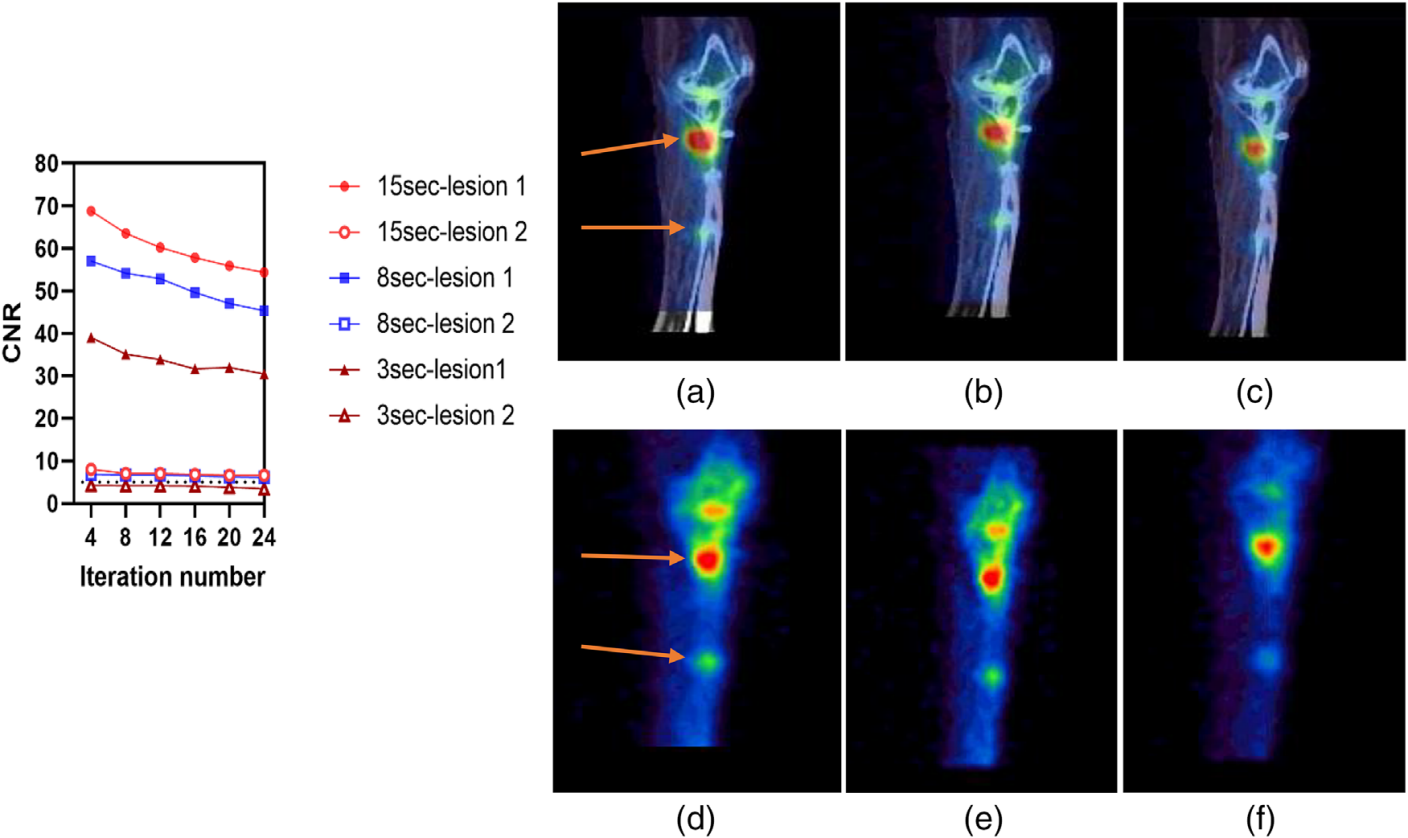
Left: contrast‐to‐noise ratio (CNR) for lesion 1 and lesion 2 for the first clinical case at different acquisition time. Right: single‐photon emission computed tomography/computed tomography (SPECT/CT) and sagittal SPECT images at: (a and d) 15 s, (b and e) 8 s, and (c and f) 3 s. The dotted line on the graph represents CNR = 5 (Rose criteria). All reconstructions used 12 iterations, eight subsets, and an 8 mm Gaussian filter

**FIGURE 10:**
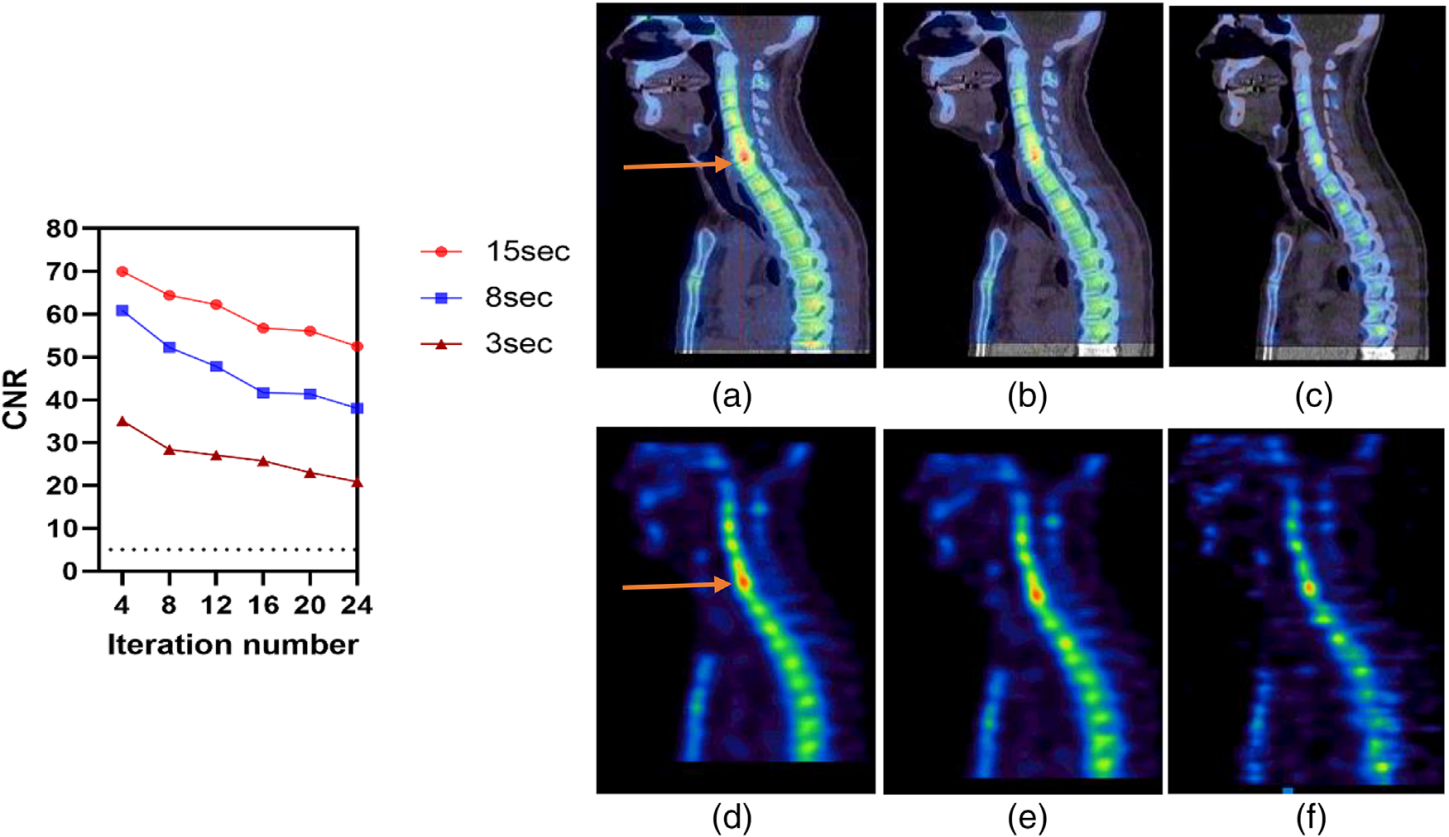
Left: contrast‐to‐noise ratio (CNR) for the abnormal uptake for the second clinical case at different acquisition time. Right: single‐photon emission computed tomography/computed tomography (SPECT/CT) and sagittal SPECT images at: (a and d) 15 s, (b and e) 8 s, and (c and f) 3 s. The dotted line on the graph represents CNR = 5 (Rose criteria). All reconstructions used 12 iterations, eight subsets, and an 8 mm Gaussian filter

Two lesions were identified in patient 1, located in the right fibular head and right tibiae. A single lesion was identified in patient 2 in the Cervical 5/6 and Cervical 6/7 areas. Figures 8, 9, 10 illustrate the contrast, noise, and CNR values for ^99m^Tc SPECT/CT clinical data acquired at various acquisition times based on ROIs drown in both abnormal and normal adjacent areas of bone. As expected, the contrast increased with an increasing number of iterations at the expense of a high noise level. ​Moreover, the noise decreased when acquisition times became longer. The convergence of the contrast was achieved at 12 iterations at all acquisition times. CNR improvement with increased acquisition time was observed for the selected abnormal lesions with CNR > 5. However, lesion 2 in patient 1 had a mild uptake in the right tibiae, and visibility dropped markedly with the shortest scan time of 3 s, reflecting the degradation of image quality as noise levels increased. Therefore, the clinical findings are in agreement with phantom results.

## 4 DISCUSSION

The purpose of this phantom and clinical study was to investigate the impact of reduced acquisition time on ^99m^Tc SPECT image quality. Optimal OSEM reconstruction parameters for reduced acquisition time data were also investigated. A NEMA image quality phantom simulated lesions of different dimensions and the impact of 4–24 iterations and 0–12 mm FWHM Gaussian filter on sphere RC and BV. The Rose criterion (CNR > 5) was used to assess sphere visibility.

RC is indicative of the extent to which the anticipated contrast among the sphere and background activity is “recovered” by the reconstructed image. As expected, the spheres were more clearly defined as acquisition time increased and count statistics improved. The overall RC increased with an increasing number of iterations at the expense of high noise. RC convergence was achieved after eight iterations, with convergence stability at all acquisition times being observed from 12 iterations, as shown in Figure 2. Nevertheless, due to the OSEM algorithm's convergence at a particular number of iterations, for example, in the present study ≥12 iteration, the contrast in the regions remained about the same (less than 3.5% variation), but the noise level increased. Matsutomo et al.^32^ reported that, in the case of a dopamine transporter SPECT, 90 was the ideal subset *×* iteration “update number”. A similar finding by Dickson et al.^33^ reported that image quality and quantification precision were enhanced when an “update number” of 100 was used with an OSEM. Despite the differences in terms of the phantom and reconstruction algorithms employed, the findings of the present work are broadly in line with these two studies.

As expected, the RC increased with increasing sphere diameter. In the present work, we found similar RC values at 15 and 8 s but lower values at 3 s. It is the detectability of the smallest lesions that are of most interest when reducing scan time. The larger lesions will be visible even at very low scan times but this is not necessarily true of smaller lesions. For example, RC values for the 10 and 37 mm spheres at 15 s/view 10.3% and 62.3%, at 8 s/view 10.3% and 61.5%, and at 3 s/view 7.6% and 57.4%, respectively. Thus, sphere detectability was better for larger spheres, consistent with the larger expected partial‐volume effect for smaller spheres arising from the finite spatial resolution of the imaging modality.^34^, ^35^, ^36^

BV reflects the image noise level. The impact of iteration number on image noise at various acquisition times is illustrated in Figure 2. Such findings are unsurprising, as the image noise is known to increase with increase iteration numbers.^37^, ^38^ Nevertheless, this work significantly revealed that shorter acquisition time was associated with a considerable increase in BV at 3 s/view (Figure 4). Therefore, a higher noise level was associated with short acquisition time leading to a reduce CNR value.

For every acquisition time, irrespective of the number of iterations, a reduction in Gaussian filter FWHM led to an increase in RC and BV. Therefore, the optimal Gaussian filter must be chosen with consideration to the trade‐off between image contrast and BV. In this study, the noise was not affected by the application of a 0–4 mm post‐filter as the pixel size of the image (4.8 mm) was larger than the value of the FWHM, which could not sufficiently suppress the noise and remove Gibbs ring artifact in the images (Figure 3). Using a higher post‐filter of 8 mm FWHM permits artifact mitigation; however, the image contrast is reduced.^39^ An FWHM Gaussian filter of 12 mm was investigated for further comparison, revealing that RC and image clarity decreased (Figure 6). This study suggests the possibility to perform acquisition times of 8 s per projection angle with total scan time 8 min per bed position (∼50% faster than the local acquisition time protocol), reconstructed with a 3D OSEM algorithm using 12 iterations and eight subsets, with a post‐Gaussian filter of 8 mm allows an optimal balance to be reached between noise and contrast (Figures 3 and 8). Nevertheless, a clinical reader study must be conducted to confirm the findings as the physician reader preferences also play a part in determining the optimal balance.

A recent prospective comparison study sought to demonstrate that BM evaluation could be effectively undertaken via the proposed 3‐min UF‐SPECT/CT acquisition of a single AFOV SPECT/CT.^1^ In their study, a standard acquisition protocol of 32 views at 20 s/view was shortened to 16 views at 10 s/view. It was demonstrated that their UF‐SPECT/CT can be performed as a clinically useful adjunct to WB planar scintigraphy without compromising diagnostic confidence. Despite the relevance of this study to our present work, there are some important differences: namely that they did not seek to optimize the reconstruction algorithm parameters, which we feel is essential when making such a significant change to the acquisition protocol, and their UF‐SPECT/CT protocol was employed as a single AFOV SPECT/CT complementary to the WB planar scan, whereas our ultimate aim is to replace the planar scan altogether with WB‐SPECT/CT alone.^3^

Clinically, detection of lesions of smaller size is most difficult, so such spheres must be considered in the context of CNR optimization. In this work, the Rose criteria (CNR > 5) was applied as a cutoff to determine small sphere visibility (Figure 5). Acquisition times of 8 and 15 s were associated with CNR > 5 for all sphere sizes (Figure 6), but a post‐smoothing filter should be considered to suppress the noise. A study by Tsujimoto et al.^40^ evaluated the Gaussian post‐filter FWHM (4–12 mm) using NEMA body phantom. According to their findings, image quality improvement was achieved with 8 and 12 mm Gaussian filters on extending acquisition times of 50 s or more per projection angle. By contrast, our present work significantly reduced this acquisition time. Therefore, we discovered that image quality improved with eight Gaussian post‐filter on acquisition time of 8–15 s per projection angle. Moreover, the two smallest spheres (13 and 10 mm) were invisible at 3 s/view as they did not conform to the Rose criterion of CNR > 5. Thus, the 3 s/view acquisition times were associated with a significant decline in lesion identification, suggesting that an increase in noise levels lowered lesion detection and, therefore, image quality. In addition, at a shorter acquisition time, the distortion of sphere shape became more obvious and took the form of an irregular “star shape” as the spheres blended with the noisy background (Figure 6). Such findings are consistent with those of earlier studies.^6^, ^41^

### 4.1 Clinical imaging

More a confirmation of the reconstruction and acquisition parameters as indicated by the phantom analysis, ^99m^Tc SPECT/CT was performed on two patients with different diagnoses and different scan areas. The first clinical case concerns a 76‐year‐old female patient who had two lesions in the right fibular head and right tibiae. Figure 8 demonstrates that the CNR for lesion 1 was associated with a CNR > 5 for all acquisition times. However, lesion 2 was not visible at 3 s projection duration and did not conform to the Rose criterion of CNR > 5. The second clinical case concerns a 55‐year‐old male patient who had osteoplastic activity in the Cervical 5/6 and Cervical 6/7. The focal abnormal lesion was associated with a CNR > 5 for all acquisition times. Therefore, the data suggest that active bone lesions will be visible even at very low scan times but this is not necessarily true of lower uptake lesions or degenerative change diseases. Hence, SPECT/CT acquired with 3 s projection duration may demonstrate difficulties in detecting such lesions. An in‐depth clinical assessment must be conducted in the future to confirm the findings and the suitability of the proposed approach for WB‐SPECT/CT.

Due to time and access constraints, this phantom study has some limitations associated with it that may be explored further in future work. The phantom analysis was performed on a sphere‐to‐background concentration ratio of 8.5:1, whereas clinically, we may expect a large amount of intra‐ and interpatient variation. Differing sphere‐to‐background ratios were not investigated in this study but would be a valuable addition to explore how recommended acquisition and reconstruction parameters may differ for varying lesion‐to‐background contrast. We Investigated the impact of acquisition time and reconstruction parameters on a single type of SPECT/CT scanner, however the same examinations repeated on other SPECT/CT devices would be an interesting exercise in the future to confirm that the proposed approach applies to any SPECT/CT scanner. Lastly, a very limited number of patients were included in this study. Therefore, a larger clinical study is required to assess acquisition and reconstructions by specialist review and comparison. Such a review is currently in progress.

## 5 CONCLUSION

The findings of this study serve as the basis for optimizing acquisition times for clinical ^99m^Tc WB‐SPECT/CT bone scans, making scan times clinically feasible and manageable for patients without compromising clinical accuracy. Optimization of reconstruction parameters requires a careful balance of image reconstruction convergence against noise levels when acquisition time is reduced. This study suggests acquisition times of 8 s per projection angle with total scan time 8 min per bed position (∼50% faster than the local acquisition time protocol), reconstructed with a 3D OSEM algorithm using 12 iterations and eight subsets, with a post‐Gaussian filter of 8 mm allows an optimal balance to be reached between noise and contrast. These conditions maintain image quality and might allow for improved patient throughput and clinical workflow.

## AUTHOR CONTRIBUTIONS

All authors participated in the study design. Mansour M. Alqahtani is the corresponding first author who performed the experimental work, analyzed and interpreted the data, and did the writing work. Peter L. Kench is the principal investigator and was involved in all stages of this project: the experimental procedure, the analysis of data, the interpretation of data, and reviewing of this manuscript. Kathy P. Willowson supported and suggested for experimental work and provided helpful feedback and comments on the manuscript. Chris Constable suggested a useful structure for the image reconstruction and provided helpful comments for the manuscript. Roger Fulton revised all equations used and provided helpful comments and suggestions for the manuscript. All authors read and approved the final manuscript.

## CONFLICT OF INTEREST

Chris Constable works for HERMES Medical Solutions. No other potential conflicts of interest relevant to this article exist.

## ETHICAL APPROVAL

This study was approved by the Northern Sydney Local Health District Human Research Ethics Committee (approval no. 2020‐02150).

## Supporting information

Supporting Information

Supporting Information

Supporting Information
