# Supplementary material for: Optimization of 99mTc whole‐body SPECT/CT image quality: A phantom study
Source: J Appl Clin Med Phys. 2022 Jan 20;23(4):e13528. doi: 10.1002/acm2.13528 (PMC8992937; doi:10.1002/acm2.13528)
Supplement: Supplementary file 1 — Supporting Information [file ACM2-23-e13528-s002.pdf]

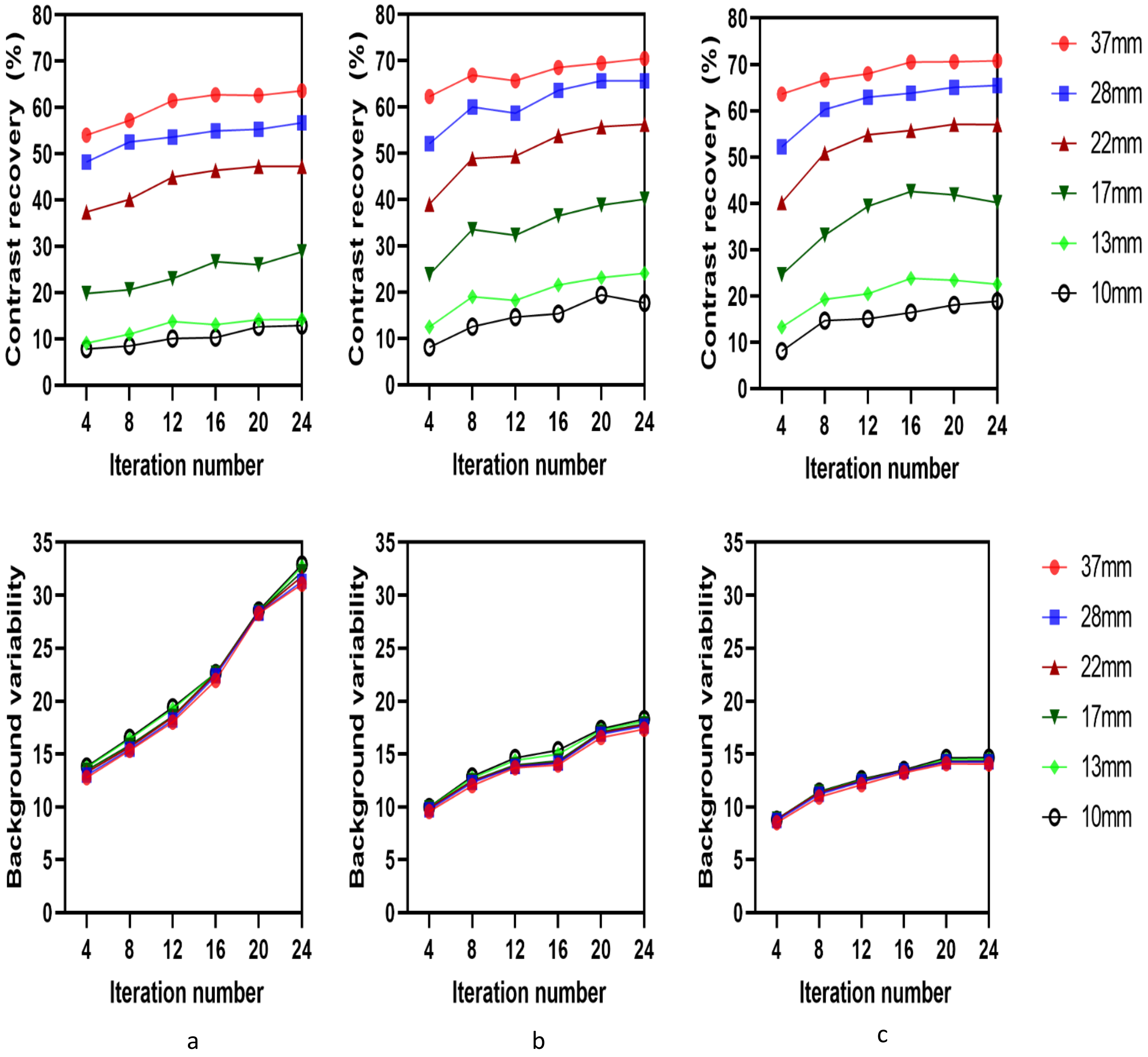

Supplemental material Fig. S1. Contrast recovery and background variability as function of iteration number. Column (a) is at 3 sec/view, (b) 8 sec/view and (c) 15sec/view. 8 subsets and without an FWHM Gaussian filter were used for all reconstructions.
