# Supplementary material for: Optimization of 99mTc whole‐body SPECT/CT image quality: A phantom study
Source: J Appl Clin Med Phys. 2022 Jan 20;23(4):e13528. doi: 10.1002/acm2.13528 (PMC8992937; doi:10.1002/acm2.13528)
Supplement: Supplementary file 3 — Supporting Information [file ACM2-23-e13528-s001.docx]

Supplementary materials:

*Supplementary material Table S1*. Contrast recovery and background variability in percent obtained for all spheres of the IEC phantoms at different acquisition time with 4-24 OSEM iterations 8 subsist and 8mm Gaussian filter were kept constant.

| **Acquisitio n time** | Contrast recovery (%) | | | | | | | Background variability | | | | | |
| --- | --- | --- | --- | --- | --- | --- | --- | --- | --- | --- | --- | --- | --- |
|  | **Spheres diameters (mm)** | | | | | | | **Spheres diameters (mm)** | | | | | |
| **3sec/view** | **Numbers of Iterations** | **10** | **13** | **17** | **22** | **28** | **37** | **10** | **13** | **17** | **22** | **28** | **37** |
|  | **4** | 5.83 | 7.96 | 16.74 | 32.79 | 43.78 | 52.96 | 11.38 | 11.31 | 10.97 | 10.82 | 10.62 | 10.42 |
|  | **8** | 6.8 | 8.85 | 17.58 | 33.48 | 44.38 | 53.46 | 12.97 | 12.86 | 12.81 | 12.66 | 12.63 | 12.6 |
|  | **12** | 7.6 | 9.39 | 22.48 | 40.12 | 47.98 | 57.48 | 13.65 | 13.56 | 13.47 | 13.34 | 13.33 | 12.94 |
|  | **16** | 7.97 | 9.96 | 23.01 | 43.12 | 49.9 | 57.34 | 15.84 | 15.71 | 15.66 | 15.46 | 15.39 | 15.35 |
|  | **20** | 7.47 | 9.47 | 21.41 | 41.42 | 48.74 | 56.46 | 17.68 | 17.61 | 17.55 | 17.49 | 17.49 | 17.47 |
|  | **24** | 8.0 | 9.64 | 23.82 | 41.19 | 49.23 | 58.7 | 18.63 | 18.53 | 18.42 | 18.39 | 18.38 | 18.36 |
|  |  | | | | | | | | | | | | |
| **8 sec/view** | **Numbers of Iterations** | **10** | **13** | **17** | **22** | **28** | **37** | **10** | **13** | **17** | **22** | **28** | **37** |
|  | **4** | 5.9 | 8.65 | 19.01 | 33.1 | 46.51 | 57.34 | 8.19 | 8.18 | 8.15 | 8.11 | 8.01 | 7.84 |
|  | **8** | 9.69 | 12.85 | 25.82 | 40.33 | 51.96 | 59.71 | 9.6 | 9.59 | 9.58 | 9.56 | 9.5 | 9.32 |
|  | **12** | 10.39 | 14.4 | 29 | 43.4 | 54.59 | 61.59 | 9.72 | 9.7 | 9.66 | 9.63 | 9.57 | 9.38 |
|  | **16** | 11.37 | 14.71 | 29.26 | 44 | 55.43 | 62.4 | 10.2 | 10.19 | 10.16 | 10.13 | 10.04 | 9.84 |
|  | **20** | 11.6 | 15.44 | 30 | 45.44 | 56.5 | 63.11 | 10.27 | 10.24 | 10.17 | 10.15 | 10.05 | 9.87 |
|  | **24** | 11.0 | 16.25 | 32.32 | 45.7 | 56.26 | 63.13 | 10.89 | 10.73 | 10.52 | 10.46 | 10.38 | 10.17 |
|  |  | | | | | | | | | | | | |
| **15sec/view** | **Numbers of Iterations** | **10** | **13** | **17** | **22** | **28** | **37** | **10** | **13** | **17** | **22** | **28** | **37** |
|  | 4 | 5.64 | 9.52 | 19.42 | 34.18 | 46.17 | 58.12 | 7.39 | 7.39 | 7.35 | 7.3 | 7.19 | 7.01 |
|  | 8 | 9.17 | 13.82 | 27.08 | 41.54 | 52.26 | 62.26 | 8.27 | 8.28 | 8.28 | 8.24 | 8.13 | 7.94 |
|  | 12 | 10.33 | 15.46 | 30.45 | 44.48 | 54.54 | 62.37 | 8.32 | 8.31 | 8.3 | 8.27 | 8.17 | 7.95 |
|  | 16 | 11.65 | 15.77 | 30.25 | 46.18 | 55.72 | 63.12 | 8.36 | 8.35 | 8.33 | 8.29 | 8.19 | 8.01 |
|  | 20 | 11.54 | 16.75 | 32.68 | 46.72 | 56.24 | 63.75 | 8.48 | 8.47 | 8.45 | 8.42 | 8.33 | 8.14 |
|  | 24 | 12.37 | 16.56 | 32.14 | 47.67 | 57.07 | 64.0 | 8.56 | 8.58 | 8.61 | 8.6 | 8.53 | 8.35 |

*Supplementary material Table S2.* Contrast recovery and background variability in percent obtained for all spheres of the IEC phantoms at different acquisition time with 4-24 OSEM iterations, 8 subsist and without postfiltering.

| **Acquisition time** | Contrast recovery (%) | | | | | | | Background variability | | | | | |
| --- | --- | --- | --- | --- | --- | --- | --- | --- | --- | --- | --- | --- | --- |
|  | **Spheres diameters (mm)** | | | | | | | **Spheres diameters (mm)** | | | | | |
| **3sec/view** | **Numbers of Iterations** | **10** | **13** | **17** | **22** | **28** | **37** | **10** | **13** | **17** | **22** | **28** | **37** |
|  | **4** | 7.82 | 9.12 | 19.81 | 37.44 | 48.23 | 54.01 | 13.81 | 13.72 | 13.45 | 13.25 | 12.98 | 12.76 |
|  | **8** | 8.48 | 11 | 20.63 | 40.15 | 52.56 | 57.23 | 16.53 | 16.41 | 16.12 | 15.95 | 15.75 | 15.61 |
|  | **12** | 10.9 | 13.74 | 23.87 | 44.99 | 53.62 | 61.47 | 19.41 | 19.24 | 18.57 | 18.49 | 18.22 | 18.02 |
|  | **16** | 10.33 | 13.12 | 26.73 | 46.46 | 54.94 | 62.78 | 22.66 | 22.66 | 22.65 | 22.55 | 22.4 | 21.95 |
|  | **20** | 12.62 | 14.18 | 26.05 | 47.28 | 55.27 | 62.61 | 28.58 | 28.42 | 28.43 | 28.4 | 28.31 | 28.29 |
|  | **24** | 12.88 | 14.25 | 28.84 | 47.31 | 56.73 | 63.64 | 32.9 | 32.71 | 32.2 | 31.78 | 31.3 | 31.05 |
|  |  | | | | | | | | | | | | |
| **8 sec/view** | **Numbers of Iterations** | **10** | **13** | **17** | **22** | **28** | **37** | **10** | **13** | **17** | **22** | **28** | **37** |
|  | **4** | 8.12 | 12.49 | 23.81 | 39.01 | 52.12 | 62.3 | 10 | 9.97 | 9.91 | 9.86 | 9.74 | 9.54 |
|  | **8** | 12.58 | 19.04 | 33.55 | 48.87 | 60.06 | 66.9 | 12.87 | 12.72 | 12.47 | 12.4 | 12.29 | 12 |
|  | **12** | 14.6 | 18.23 | 32.27 | 49.44 | 58.69 | 65.71 | 14.63 | 14.39 | 13.95 | 13.85 | 13.81 | 13.68 |
|  | **16** | 15.38 | 21.56 | 36.53 | 53.84 | 63.64 | 68.57 | 15.36 | 14.98 | 14.33 | 14.18 | 14.13 | 13.96 |
|  | **20** | 19.42 | 23.16 | 38.8 | 55.74 | 65.7 | 69.52 | 17.36 | 17.24 | 17.08 | 17 | 16.86 | 16.56 |
|  | **24** | 17.73 | 24.08 | 40.11 | 56.32 | 65.73 | 70.48 | 18.29 | 18.1 | 17.84 | 17.76 | 17.64 | 17.34 |
|  |  | | | | | | | | | | | | |
| **15sec/view** | **Numbers of Iterations** | **10** | **13** | **17** | **22** | **28** | **37** | **10** | **13** | **17** | **22** | **28** | **37** |
|  | **4** | 8.11 | 13.25 | 24.62 | 40.19 | 52.21 | 63.62 | 8.78 | 8.82 | 8.86 | 8.83 | 8.72 | 8.5 |
|  | **8** | 14.66 | 19.24 | 33.14 | 50.87 | 60.29 | 66.65 | 11.45 | 11.43 | 11.4 | 11.34 | 11.19 | 10.91 |
|  | **12** | 15.07 | 20.45 | 39.34 | 54.82 | 62.91 | 68 | 13.48 | 13.41 | 13.39 | 13.41 | 13.38 | 13.22 |
|  | **16** | 16.41 | 23.82 | 42.53 | 55.75 | 63.78 | 70.53 | 13.82 | 13.72 | 13.63 | 13.56 | 13.44 | 13.3 |
|  | **20** | 18.1 | 23.42 | 41.83 | 57.06 | 65.03 | 70.59 | 14.64 | 14.49 | 14.32 | 14.29 | 14.25 | 14.06 |
|  | **24** | 18.9 | 22.55 | 40.16 | 57.03 | 65.49 | 70.78 | 14.66 | 14.51 | 14.33 | 14.28 | 14.24 | 14.05 |

*Supplementary material Table S3.* Contrast recovery and background variability in percent obtained for all spheres of the IEC phantoms at different acquisition time with 0-12mm Gaussian filter and 12 titration and 8 subsist were kept constant.

| **Acquisition time** | Hot contrast recovery (%) | | | | | | | Background variability | | | | | |
| --- | --- | --- | --- | --- | --- | --- | --- | --- | --- | --- | --- | --- | --- |
|  | **Spheres diameters (mm)** | | | | | | | **Spheres diameters (mm)** | | | | | |
| **3sec/view** | **Gaussian filter** | **10** | **13** | **17** | **22** | **28** | **37** | **10** | **13** | **17** | **22** | **28** | **37** |
|  | **0** | 10.09 | 13.74 | 23.0 | 44.9 | 53.62 | 61.47 | 19.41 | 19.24 | 18.57 | 18.49 | 18.22 | 18.02 |
|  | **4** | 9.14 | 12.79 | 21.69 | 42.75 | 50.15 | 57.05 | 18.39 | 18.35 | 18.27 | 18.17 | 17.96 | 17.79 |
|  | **8** | 7.64 | 9.39 | 22.18 | 40.12 | 47.98 | 57.48 | 13.65 | 13.56 | 13.47 | 13.34 | 13.33 | 12.94 |
|  | **12** | 7.20 | 8.0 | 21.14 | 39.74 | 44.30 | 55.69 | 11.66 | 11.36 | 11.29 | 11.17 | 11.09 | 11.02 |
|  |  | | | | | | | | | | | | |
| **8 sec/view** | **Gaussian filter** | **10** | **13** | **17** | **22** | **28** | **37** | **10** | **13** | **17** | **22** | **28** | **37** |
|  | **0** | 14.6 | 18.23 | 32.27 | 49.44 | 58.69 | 65.71 | 14.63 | 14.39 | 13.95 | 13.85 | 13.81 | 13.68 |
|  | **4** | 13.48 | 19.42 | 33.86 | 48.71 | 58.56 | 63.89 | 13.77 | 13.68 | 13.59 | 13.43 | 13.39 | 13.34 |
|  | **8** | 10.39 | 14.4 | 29 | 43.4 | 54.59 | 61.59 | 9.72 | 9.7 | 9.66 | 9.63 | 9.57 | 9.38 |
|  | **12** | 6.68 | 8.93 | 20.92 | 32.20 | 43.63 | 51.75 | 7.27 | 7.23 | 7.19 | 7.15 | 7.04 | 7.03 |
|  |  |  |  |  |  |  | | | | | | | |
| **15 sec/view** | **Gaussian filter** | **10** | **13** | **17** | **22** | **28** | **37** | **10** | **13** | **17** | **22** | **28** | **37** |
|  | **0** | 15.07 | 20.45 | 39.34 | 54.82 | 62.91 | 68 | 13.41 | 13.39 | 13.41 | 13.38 | 13.22 | 13.41 |
|  | **4** | 14.06 | 19.08 | 36.72 | 51.17 | 58.72 | 63.42 | 12.62 | 12.52 | 12.46 | 12.45 | 12.38 | 12.1 |
|  | **8** | 10.33 | 15.46 | 30.45 | 44.48 | 54.54 | 62.37 | 8.32 | 8.31 | 8.3 | 8.27 | 8.17 | 7.95 |
|  | **12** | 6.51 | 10.05 | 21.64 | 33.18 | 43.84 | 53.01 | 6.1 | 6.13 | 6.11 | 6.07 | 5.99 | 5.84 |
